# Paediatric parapneumonic effusion – a twenty-year clinical narrative

**DOI:** 10.1007/s15010-025-02662-1

**Published:** 2025-10-14

**Authors:** Leonie Bregy, Philipp K.A. Agyeman, Andrea Duppenthaler, Elisabeth Kieninger, Matthias Horn, Jonathan Juzi, Dietmar Cholewa, Carmen Casaulta, Matthias V. Kopp, Christoph Aebi, Nina Schöbi

**Affiliations:** 1 Division of Paediatric Infectious Disease, Department of Paediatrics, Bern University Hospital, Inselspital, University of Bern, Bern, CH-3010 Switzerland; 2 Division of Paediatric Respiratory Medicine and Allergology, Department of Paediatrics, Bern University Hospital, Inselspital, University of Bern, CH-3010 Bern, Switzerland; 3 Department of Paediatrics, Bern University Hospital, Inselspital, University of Bern, CH-3010 Bern, Switzerland; 4 Department of Paediatric Surgery, Bern University Hospital, Inselspital, University of Bern, CH-3010 Bern, Switzerland; 5 Airway Research Center North (ARCN), University of Lübeck, D-23562 Lübeck, Germany

**Keywords:** Parapneumonic effusion, Pleural empyema, Hospitalisation, VATS, Pleural drainage, GAS

## Abstract

**Purpose:**

Paediatric parapneumonic effusion (PPE) is accompanied by an increased risk of complications, e.g., sepsis or lung sequelae. Treatment strategies span from antibiotics alone to surgical interventions, but an internationally accepted guideline is lacking. With this study, we aim to better understand how management strategies influence short-term outcome parameters, like length of stay, antibiotic treatment duration, and lung damage.

**Methods:**

Retrospective observational single-centre study. Patients admitted from 1 July 2004 to 30 June 2024 with PPE to our tertiary hospital were analysed. We used the exact Jonckheere-Terpstra test to analyse trends over time.

**Results:**

A total of 278 patients were included, 23 (8%) had to be excluded for lack of informed consent. A majority (173/255, 68%) were treated with pleural drainage. Over time, drains were increasingly more often inserted without surgery, 20% vs. 65% (*p* = 0.001) in 2004–2008 vs. 2020–2024. Intravenous antibiotic treatment duration declined from 15 days in 2004–2008 to 11 days in 2020–2024, *p* = 0.002. The most commonly identified pathogen was *S. pneumoniae* (39%), followed by *S. pyogenes* (18%). *S. pyogenes* compared to *S. pneumoniae* was more often associated with sepsis or toxic shock (45% vs. 6%, *p* < 0.0001), but fewer patients showed radiologic evidence for acute lung damage (68% vs. 23%, *p* < 0.001).

**Conclusion:**

We found considerable clinical differences in patients with PPE caused by *S. pneumoniae* vs. *S. pyogenes.* The former was associated with substantially greater lung damage.

**Supplementary Information:**

The online version contains supplementary material available at 10.1007/s15010-025-02662-1.

## Introduction

Paediatric parapneumonic effusion (PPE), complicating community-acquired pneumonia, causes significant morbidity, mostly in children under the age of 5 years. Even though numbers declined with the introduction of the 13-valent pneumococcal vaccine, different groups reported increasing case frequencies of PPE over the last decade [1–6]. In Europe, the most common organisms are *S. pneumoniae*, followed by *S. pyogenes* and *S. aureus.* [7] However, pathogen identification is often hampered either because of negative test results or because appropriate specimens for microbiological testing are lacking. Patients with PPE are at increased risk for systemic complications, such as severe sepsis, but also for acute lung damage, such as pneumatocele or bronchopleural fistula. Overall, the approach to patients with PPE is controversial, and hospital guidelines widely differ [8–10]. Management strategies span from conservative treatment, consisting of antibiotics with or without a pleural drain, to a variety of surgical interventions, such as video-assisted thoracoscopic surgery (VATS) or open thoracotomy with decortication [11–13]. When early chest interventions are applied, fibrinolytics are sometimes administered, however, their beneficial effect is debated, and data mainly originate from the adult population [14]. Medium-term clinical and radiographic outcomes are overall good, but long-term data investigating respiratory consequences in adulthood are limited [15–18]. 

This observational study aims to better understand how periodically fluctuating management preferences at a single institution influence relevant short-term outcomes, e.g., length of stay (LoS), duration of antibiotic treatment, and structural lung damage. In addition, we sought to examine the variations in clinical presentation depending on the causative organism.

## Methods

### Setting and data retrieval

This is a retrospective, single-centre, non-interventional, clinical study of cases of PPE from 1 July 2004 to 30 June 2024 conducted at the Department of Paediatrics, Bern University Hospital, Inselspital, University of Bern, Switzerland. This institution is the sole paediatric in-patient service provider for a general population of 1.1 million, including approximately 200’000 individuals below 18 years of age [19]. The case identification process consisted of (1) an electronic search of the medical records for the International Disease Classification (ICD)-9/10 codes J18, J86, J90, and J94 performed by the hospital’s coding office, (2) an electronic text search of the list of discharge diagnoses (terms: parapneumonic effusion; pleural empyema, pleuropneumonia, complicated pneumonia), and (3) a query of the electronic clinical microbiology database for pleural fluid samples testing positive for bacterial species by culture, Polymerase Chain Reaction (PCR), or antigen testing. The clinical variables, as listed in Table 1, were extracted from the medical record of each case.

**Table 1:** Clinical characteristics of 255 patients with parapneumonic effusion (PPE), Department of Paediatrics, Bern University Hospital in 2004–2024

	with drainage	no drainage		*p* value (univariate)
	n (%)/median [IQR]	n (%)/median [IQR]		
Patients	173 (68)	82 (32)		
Female sex	78 (45)	34 (41)		0.68
Age (years)	4.1 [2.4–6.7]	4.7 [2.6–7.4]		0.37
Secondary referral from peripheral hospital	65 (38)	12 (15)		< 0.001
Prior oral antibiotic therapy	50 (29)	31 (38)		0.20
Comorbidity, any	33 (19)	11 (13)		0.35
Respiratory	16 (9)	2 (2)		0.09
Neurologic	8 (5)	2 (2)		0.51
Down syndrome	2 (1)	3 (4)		0.33
Prematurity < 37 weeks	1 (1)	2 (2)		0.24
Other	6 (3)	2 (2)		0.73
Admitting diagnoses				
Pneumonia with right-sided PPE	70 (40)	41 (50)		0.19
Pneumonia with left-sided PPE	86 (50)	36 (44)		0.46
Pneumonia with bilateral PPE	17 (10)	5 (6)		0.45
Clinical scarlet fever	14 (8)	5 (6)		0.75
Varicella	2 (1)	2 (2)		0.47
Acute otitis media	21 (12)	7 (9)		0.52
Septic shock or toxic shock syndrome	19 (11)	4 (5)		0.18
Haemolytic-uraemic syndrome	7 (4.0)	1 (1)		0.28
CRP, admission (mg/L)^1,3^	267 (103)	230 (112)		< 0.01
WBC, admission (G/)^1,3^	16.9 (9.5)	17.9 (10.2)		0.44
Platelet count, highest (G/L)^1,3^	827 (329)	607 (300)		< 0.0001
Bacteraemia	21 (12)	9 (11)		0.84
Radiologic evidence for				
Pneumatocele or bullae	53 (31)	5 (6)		^2^
Seropneumothorax	53 (31)	0		^2^
Bronchopleural fistula	28 (16)	0		^2^
Intrapulmonary abscess	13 (8)	1 (1)		^2^
Necrotising pneumonia	24 (14)	7 (9)		^2^
Microbiology				
*Streptococcus pneumoniae*	68 (39)	7 (9)		^2^
*Streptococcus pyogenes*	31 (18)	3 (4)		^2^
Other	12 (7)	2 (2)		^2^
No pathogen identified	62 (36)	63 (77)		^2^
***Interventions and outcomes***				
Length of stay (days)	15.0 [12.0–21.0]	9.0 [5.3–12.0]		< 0.0001
Antibiotic therapy duration (days)				
Intravenous	14.0 [11.0–19.0]	8.0 [5.0–11.0]		< 0.0001
Intravenous plus oral step-down	15.0 [14.0–22.0]	14.0 [10.0–14.0]		< 0.0001
PICU admission	81 (47)	7 (8.5)		< 0.0001
Length of stay in PICU (days)	3.0 [2.0–6.0]	3.0 [2.0–3.0]		0.23
Inotrope support	20 (12)	1 (1)		0.010
Intubation	22 (13)	0		< 0.01
Rehospitalisation for complications of PPE	12 (7)	4 (5)		0.72
Death	1 (1)	0		

^1^ mean and standard deviation (SD) are shown for this variable

^2^ no statistical analysis performed, as the extent of investigations performed differed substantially between groups

^3^ variables were normally distributed

### Case definition and inclusion/exclusion criteria

Cases were included, if the patient was admitted to the in-patient service of our hospital during the designated observation period, was below 18 years of age on the day of admission, and presented with all of the following clinical criteria: (1) acute-onset respiratory disease of fewer than 30 days, (2) pulmonary parenchymal infiltrate, (3) PPE > 10 mm in width (plain chest radiograph or chest ultrasound), and (4) a maximum serum C-reactive protein ≥ 100 mg/L. The exclusion criteria included at least one of the following: (1) lack of general or study-specific written informed consent, (2) PPE associated with infection caused by *Mycobacterium spp*, *Actinomyces spp*, *Nocardia spp*, or fungi, (3) non-inflammatory pleural effusion. For patients referred from a peripheral hospital, we defined day 1 of the hospital stay as the day of admission to the peripheral hospital.

After completion of baseline analyses (Table 1), we conducted subsequent analyses only on patients treated with pleural drainage.

### Microbiology

Bacterial pathogens cultured from blood or pleural fluid or identified by broad-spectrum PCR of pleural fluid were considered as the microbial aetiology of the respective PPE. If broad-spectrum PCR could not differentiate between *S. pneumoniae* and other members of the *Streptococcus mitis* group (e.g., *Streptococcus pseudopneumoniae*), *S. pneumoniae* was considered as the aetiologic agent [20]. 

### Radiology

Variables of interest were extracted from the written radiology reports of chest examinations and included the terms bullae, pneumatocele, seropneumothorax, fistula, abscess, necrosis, and necrotising pneumonia. Lung damage was defined as the radiological presence of at least one of the five following critieria, i.e., pneumatocele or bullae, seropneumothorax, bronchopleural fistula, intrapulmonary abscess, or necrotising pneumonia.

### Statistical analysis

For analysis of trends over time, the 20-year study period was arbitrarily divided into five periods of four epidemiologic years each. Data are presented as frequencies and proportions for categorical variables and as medians with interquartile ranges (IQR) or means with standard deviations (SD) for continuous variables. Categorical variables were compared with the χ2 test or Fisher Exact test. Continuous variables were compared using non-parametric tests (Mann–Whitney U-test or the Kruskal-Wallis test) or parametric tests (t-test, analysis of variance [ANOVA]). Correlations between continuous variables were examined using linear regression analysis. For comparison of PPE caused by *S. pneumoniae* or *S. pyogenes*, we fitted a logistic regression model for potential associations of the pathogen detected and age, sex, bacteraemia, highest platelet count, type of intervention, LoS, and septic shock (operationalised as the need for inotropes). We separately fitted univariable models and a multivariable (adjusted) model containing all potentially associated variables. We used the exact Jonckheere-Terpstra (JT) test to analyse trends over time of continuos data with 10,000 permutations. A statistical significance level of 5% was considered statistically significant throughout.

R software package, version 4.0.3 (Vienna, Austria; https://www.R-project.org/), VassarStats (http://www.vassarstats.net), and GraphPad Prism version 10.0.0 for Windows (GraphPad Software, Boston, Massachusetts, USA (http://www.graphpad.com) were used for analyses and figure drawing.

## Results

Between 1 July 2004 and 30 June 2024, a total of 278 patients were admitted with PPE. Twenty-three cases (8%) were excluded for lack of written informed consent. The study cohort consisted of 255 patients, of whom 143 (56%) were male. The median age at presentation was 4.4 years [2.4–6.9]. In the majority of patients, 173/255 (68%), a chest intervention was performed (pleural drainage with or without surgical procedures). Patients with pleural drainage vs. those without were significantly more often referred from another hospital, 65/173 (38%) vs. 12/82 (15%), *p* < 0.001, and had higher inflammatory parameters (CRP at admission 267 mg/l (103) vs. 230 mg/l (112), *p* < 0.01; highest platelet count 827 G/l (329) vs. 607 G/l (300), *p* < 0.0001). Acute lung damage was more common in patients with pleural drainage compared to those without (pneumatocele, 53/173 (31%) vs. 5/82 (6%); intrapulmonary abscess, 13/173 (8%) vs. 1/82 (1%)). LoS (15.0 days [12.0–21.0] vs. 9.0 days [5.3–12.0], *p* < 0.0001), and the duration of intravenous antibiotic treatment was longer (14.0 days [11.0–19.0] vs. 8.0 days [5.0–11.0], *p* < 0.0001) in patients with a chest intervention. Table 1.

### Epidemiology – time trends

**Fig. 1:**
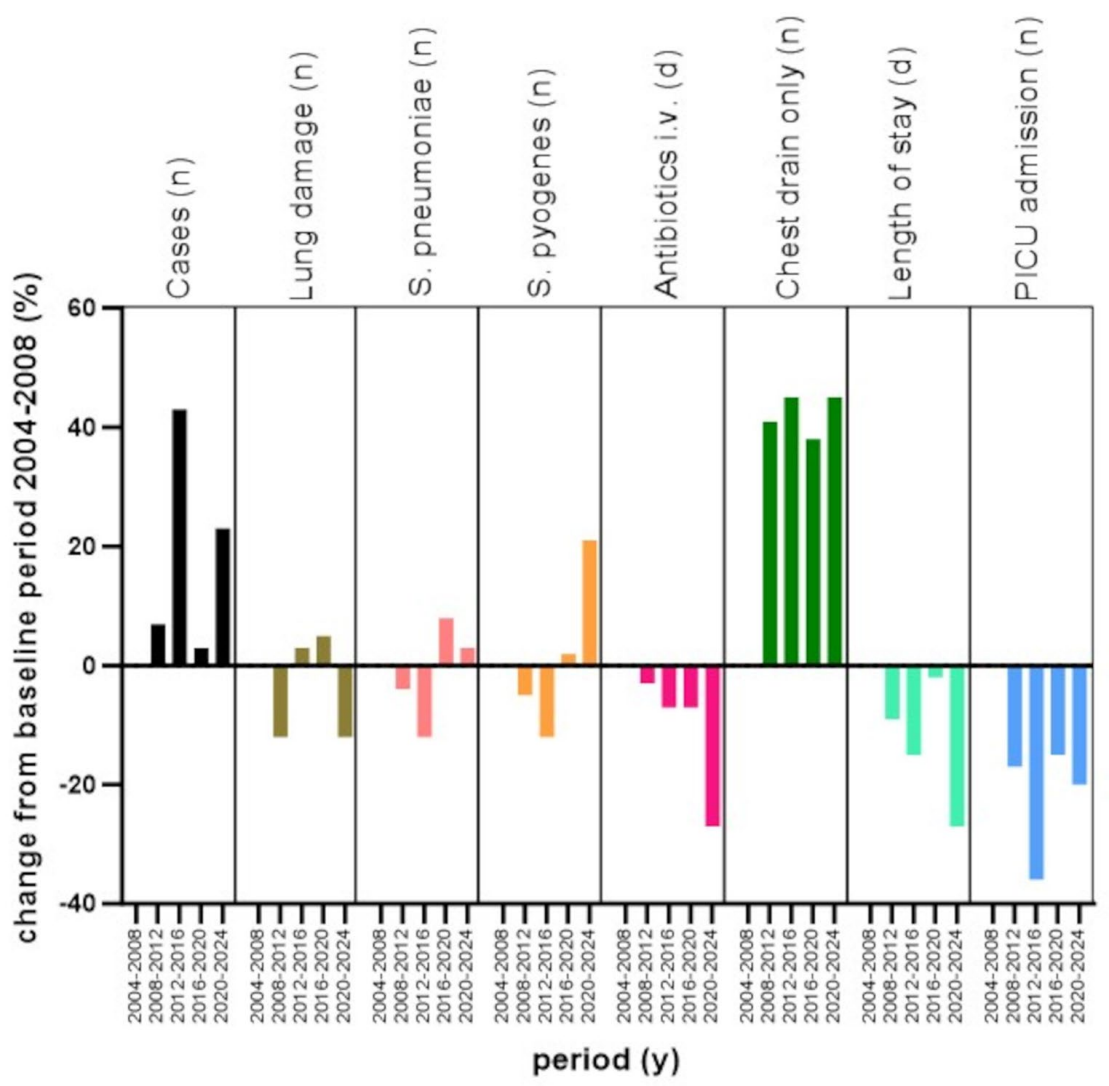
For each of the variables indicated by text labels, a datapoint for each of the 5 sequential periods shown on the x-axis is represented by a column. The data point for the 2004–2008 period is set to zero for each variable, and the data points for the remaining 4 time periods indicate the percent change compared to 2004–2008

**Fig. 2:**
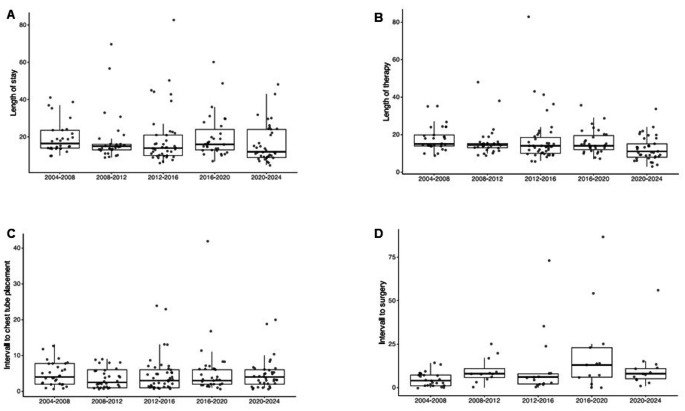
Clinical characteristics for each of the 5 sequential periods. **A**) Length of stay (LOS). The dataset contains 173 observations, 30 in 2004–2008 (median LOS 16.5 days), 32 in 2008–2012 (median LOS 15 days), 43 in 2012–2016 (median LOS 14 days), 31 in 2016–2020 (median LOS 16 days), 37 in 2020–2024 (median LOS 12 days), JT = 5248.5, *p* = 0.0562; **B**) Days on iv antibiotic treatment (LOT). The dataset contains 173 observations, 30 in 2004–2008 (median LOT 15 days), 32 in 2008–2012 (median LOT 14.5 days), 43 in 2012–2016 (median LOT 14 days), 31 in 2016–2020 (median LOT 14 days), 37 in 2020–2024 (median LOT 11 days), JT = 4634.5, *p* = 0.0008; **C**) Intervall from admission to chest tube insertion. The dataset contains 171 observations, 30 in 2004–2008 (median intervall 4 days), 32 in 2008–2012 (median intervall 2.5 days), 41 in 2012–2016 (median intervall 3 days), 31 in 2016–2020 (median intervall 3 days), 37 in 2020–2024 (median intervall 4 days), JT = 5830, *p* = 0.9868; **D**) Intervall from admission to surgery. The dataset contains 77 observations, 24 in 2004–2008 (median intervall 4 days), 12 in 2008–2012 (median intervall 8 days), 15 in 2012–2016 (median intervall 6 days), 13 in 2016–2020 (median intervall 13 days), 13 in 2020–2024 (median intervall 8 days), JT = 1413, *p* = 0.0218

We found PPE frequencies from 30 cases (2004–2008) to a maximum of 43 cases (2012–2016) per 4 years, with an overall trend of increasing frequencies. While over the entire study period, *S. pneumoniae* was the most commonly identified pathogen, 68/173 (39%), we found significantly more cases of *S. pyogenes* infection in 2020–2024, 14/37 (38%), *p* = 0.003. In 62/173 (36%) patients, we failed to identify an organism, this proportion declined to 16% in 2020–2024. The duration of intravenous antibiotic treatment decreased significantly from 15.0 days [14.0-19.8] in 2004–2008 to 11.0 days [8.0–15.0] in 2020–2024, JT = 4634.5, *p* = 0.0008. LoS declined but not significantly from 16.5 days [14.0-23.5] to 12.0 days [9.0–24.0], JT = 5248.5, *p* = 0.056. At the beginning of our observation, fewer patients were treated with a pleural drain alone compared to the most recent period, 6/30 (20%) vs. 24/37 (65%), *p* = 0.001. In 2004 to 2008, VATS was performed in 22/30 (73%) patients. Thereafter, the number of VATS dropped to a nadir in 2016–2020 of 4/31 (13%) cases before increasing again in 2020–2024 to 13/37 (35%), *p* < 0.0001. While thoracotomy/decortication was performed in less than 10% throughout the observation period, from 2016 to 2020 9/31 (29%) patients required such treatment, *p* = 0.0005. In 2008–2012, fibrinolytic therapy was used in 22/32 (67%), thereafter numbers declined to 11% in 2020–2024, *p* < 0.0001. Time from admission to chest tube insertion remained unchanged with a maximum median of 4 days in 2004–2008 vs. a minimal median of 2.5 days in 2008–2012, JT = 5830, *p* = 0.987. Time to surgery significantly differed between the periods with a minimum median interval of 4 days in 2004–2008 to a maximum median interval of 13 days in 2016–2020, JT = 1413, *p* = 0.022. Lastly, readmission rate due to complications related to PPE was low, ranging from 0 to 14%, and showed no significant differences between the periods. Table S1, Figs. 1 and 2.

### Pleural drainage alone versus surgical intervention

Of the 173 patients with a chest intervention, 96 (55.5%) had a pleural drain alone compared to 77 (44.5%) who also underwent VATS (62/77, 81%) and/or thoracotomy (16/77, 21%). In most situations, surgical intervention was performed > 2 days after admission, 60/77 (78%), compared to 12/77 (16%) within the first 24 hours. Patients who had a pleural drain alone compared to those with surgical interventions showed no differences in CRP, 262 mg/l (104 mg/l) vs. 274 mg/l (102 mg/l), *p* = 0.43, and WBC, 16.5 G/l (9.1 G/l) vs. 17.3 G/l (10.1 G/l), *p* = 0.56, at admission, but those patients showed fewer pulmonary sequelae, e.g., pneumatocele, 21/96 (22%) vs. 32/77 (42%), *p* < 0.01, or bronchopleural fistula, 9/96 (9.4%) vs. 19/77 (25%), *p* = 0.012. In patients with a pleural drain alone compared to those with a surgical intervention, the drain lasted for a median of 4.0 days [2.0–5.0] vs. 7.0 days [5.0–7.0], *p* < 0.0001. Intravenous antibiotic treatment in patients with a pleural drain alone was significantly shorter than in patients with surgical intervention, 13.0 days [9.0–14.0] vs. 16.0 days [14.0–19.0], *p* < 0.0001. Fewer patients needed to be admitted to PICU, 34/96 (35%) vs. 47/77 (61%), *p* < 0.01, with similar proportions of patients presenting with septic or toxic shock (11% vs. 10%, *p* = 1.00), requiring inotropics (11% vs. 12%, *p* = 0.84) or intubation (9.4% vs. 17%, *p* = 0.21). Lastly, LoS was shorter, 13.0 days [10.0–16.0] vs. 18.0 days [15.0–22.0], *p* < 0.0001. Table 2.

**Table 2:** Clinical characteristics in 173 patients with parapneumonic effusion receiving chest tube drainage alone vs. surgical intervention (VATS and/or thoracotomy)

	Chest tube alone	VATS and/or thoracotomy		*p* value (univariate)
	n (%)/median [IQR]	n (%)/median [IQR]		
Patients	96	77		
Female sex	41 (43)	37 (48)		0.58
Age (years)	4.1 [2.4–6.2]	4.1 [2.4–6.7]		0.93
Comorbidity, any	23 (24)	10 (13)		0.10
Clinical scarlet fever	12 (13)	2 (2.6)		< 0.01
Septic shock or toxic shock syndrome	11 (11)	8 (10)		1.00
CRP, admission (mg/L)^1,2^	262 (104)	274 (102)		0.43
WBC, admission (G/L)^1,2^	16.5 (9.1)	17.3 (10.1)		0.56
Platelet count, highest (G/L)^1,2^	752 (306)	919 (336)		< 0.01
Bacteraemia	9 (9.4)	12 (16)		0.31
Radiologic findings				
Pneumatocele or bullae	21 (22)	32 (42)		< 0.01
Seropneumothorax	19 (20)	34 (44)		< 0.01
Bronchopleural fistula	9 (9.4)	19 (25)		0.012
Intrapulmonary abscess	4 (4.2)	9 (12)		0.12
Necrotising pneumonia	6 (6.3)	18 (23)		< 0.01
Pathogen identified				
*S. pneumoniae*	33 (34)	35 (45)		0.19
*S. pyogenes*	21 (22)	10 (13)		0.19
Other	6 (6.3)	7 (9.1)		0.68
No pathogen identified	36 (38)	25 (32)		0.60
Antibiotic therapy, duration (days)				
Intravenous	13.0 [9.0–14.0]	16.0 [14.0–19.0]		< 0.0001
Intravenous plus oral step-down	14.0 [14.0-16.3]	19.0 [15.0–22.0]		< 0.0001
Chest interventions				
Chest drainage duration (days)	4.0 [2.0–5.0]	7.0 [5.0–7.0]		< 0.0001
Fibrinolytic therapy	40 (42)	17 (22)		0.010
VATS	0	62 (81)		
Thoracotomy	0	16 (21)		
Length of stay (d)	13.0 [10.0–16.0]	18.0 [15.0–22.0]		< 0.0001
PICU admission	34 (35)	47 (61)		< 0.01
Inotropes	11 (11)	9 (12)		0.84
Intubation	9 (9.4)	13 (17)		0.21
Rehospitalisation for complications of PPE	4 (4.2)	8 (10)		0.19

^1^ mean and standard deviation (SD) are shown for this variable

^2^ variables were normally distributed

### Comparison between PPE associated with S. pneumoniae and S. pyogenes in patients with a chest intervention

Next, we examined clinical differences in PPE associated with the two most commonly identified pathogens. S. *pneumoniae* was identified in 68/173 (39%) cases and *S. pyogenes* in 31/173 (18%) cases. Scarlet fever was exclusively present in patients with PPE caused by *S. pyogenes*, 11/31 (35%). Patients with PPE caused by *S. pneumoniae* significantly more often received antibiotic treatment prior to admission, 25/68 (37%) vs. 2/31 (6%), *p* < 0.001, whereas patients with *S. pyogenes* significantly more often presented with signs of septic or toxic shock syndrome, 14/31 (45%) vs. 4/68 (6%), *p* < 0.0001. No difference was found in the proportion of patients admitted to PICU. However, patients with *S. pyogenes* more often needed inotropic support, 12/31 (39%) vs. 7/68 (10%), *p* < 0.001. Evidence of lung damage was more common in *S. pneumoniae-*associated PPE, e.g., pneumatocele, 35/68 (51%) vs. 4/31 (13%), *p* < 0.001, or bronchopleural fistula, 21/68 (31%) vs. 1/31 (3%), *p* = 0.005. More frequent inotropic support associated with *S. pyogenes* and structural lung damage associated with *S. pneumoniae* were confirmed to be significant in the multivariate analysis. Table 3.

**Table 3:** Clinical characteristics in 173 patients with parapneumonic effusion and chest intervention according to the bacterial aetiology

	S. pneumoniae	S. pyogenes		Univariate analysis			Multivariate analysis	
	n (%)/median [IQR]	n (%)/median [IQR]		OR (95% CI)^1^	*p* value		adjusted OR (95% CI)^1^	*p* value
Cases (n)	68	31						
Female sex	35 (51)	12 (39)		1.679 (0.713–4.068)	0.61		0.986 (0.847–1.152)	0.85
Age (years)	3.9 [2.3–5.6]	4.1 [1.8–6.6]		1.031 (0.911–1.16)	0.24		1.461 (0.469–4.722)	0.51
Prior oral antibiotic therapy	25 (37)	2 (6)		8.430 (1.852–38.366)	< 0.001			
Comorbidity, any	12 (18)	4 (13)		1.446 (0.427–4.905)	0.76			
Clinical scarlet fever	0	11 (35)		0	< 0.0001			
Septic shock or toxic shock syndrome	4 (6.0)	14 (45)		0.076 (0.022–0.2619)	< 0.0001			
CRP, admission (mg/L)^1,3^	287 (99)	267 (121)			0.10			
WBC, admission (G/L)^1,3^	15.5 (9.0)	17.5 (11.1)			0.33			
Platelet count, highest (G/L)^1,3^	941 (326)	750 (325)		0.998 (0.996–0.999)	< 0.001		0.998 (0.996-1)	0.043
Bacteraemia	17 (25)	2 (7)		0.207 (0.031–0.791)	0.019		0.176 (0.018–0.953)	0.043
Radiographic findings								
Pneumatocele or bullae	35 (51)	4 (13)		7.159 (2.261–22.672)	< 0.001			
Seropneumothorax	34 (50)	1 (3)		30.000 (3.867-232.647)	< 0.0001			
Bronchopleural fistula	21 (31)	1 (3)		13.494 (1.712-104.933)	0.005			
Intrapulmonary abscess	2 (2.9)	4 (13)		0.205 (0.035–1.184)	0.075			
Necrotising pneumonia	14 (21)	3 (10)		0.938 (0.377–2.336)	1			
≥1 of the 5 criteria listed above	46 (68)	7 (23)		0.139 (0.049–0.357)	< 0.001		0.177 (0.036–0.705)	0.013
Antibiotic therapy, duration (days)								
Intravenous	15.0 [13.0-19.3]	14.0 [10.0-19.5]			0.06			
Intravenous plus oral step-down	15.4 [14.0-22.3]	14.0 [13.5–22.0]			0.06			
Chest interventions								
VATS and/or thoracotomy	35 (51)	10 (32)		2.227 (0.914–5.427)	0.12		1.248 (0.36–4.501)	0.73
Chest drainage duration (days)	6.0 [4.0–11.0]	4.0 [2.0–6.0]			< 0.001			
Fibrinolytic therapy	14 (21)	9 (29)		0.634 (0.240–1.67)	0.51			
Length of stay (d)	16.5 [14.0–25.0]	14.0 [10.5–20.5]		0.968 (0.925–1.004)	0.09		0.955 (0.893–1.01)	0.11
PICU admission	37 (54)	20 (66)		0.657 (0.273–1.578)	0.47			
Inotropes	7 (10)	12 (39)		5.504 (1.942–16.742)	< 0.001		25.619 (4.325-246.176)	< 0.001
Intubation	10 (15)	9 (29)		0.422 (0.151–1.175)	0.16			
Rehospitalisation for complications of PPE	5 (7.4)	3 (9.7)		0.741 (0.165–3.317)	1			

^1^ odds ratio (95% confidence interval)

^2^ mean and standard deviation (SD) are shown for this variable

^3^ variables were normally distributed

## Discussion

Retrospective clinical analyses over extended periods are subject to many confounders that can impact the results but offer valuable insights into the overall achievements of clinical management for a given disease entity. We found considerable clinical differences in patients with PPE caused by *S. pneumoniae* vs. *S. pyogenes. S. pneumoniae* was associated with substantially greater lung damage. However, despite changes in the preferred management of patients with PPE over the 20 years of observation, major outcome parameters for patients, like LoS or readmission rate related to complications, remained essentially unchanged.

Our initial data analysis yielded substantial clinical differences between patients with compared to those without chest interventions. Therefore, and because it is likely that our case search strategy identified the former more reliably than the latter, we focused subsequent analyses on patients who did undergo chest interventions and excluded patients without.

The management of patients with PPE is an unresolved debate [13]. First, it is unclear if chest interventions should be offered aggressively, in the sense of optimising source control, or should be reserved for complicated cases [8, 9]. As of today, no randomised controlled trial has addressed this question, and the available observational data are conflicting [11, 12]. Second, it is unclear if pleural drainage alone is beneficial compared to surgical management [8, 9, 21–25]. While in our cohort, only a third of patients were managed conservatively, the approach to any kind of chest intervention experienced some impressive changes over time. At the beginning of our observation, primary VATS was the preferred approach. The insertion of a pleural drain alone was performed in only a minority of patients. This changed significantly after 2008 when a ‘pleural drain alone’ strategy was favoured. From 2016 to 2020, we experienced a phase when thoracotomy plus decortication became more frequent than VATS. During this period, we found that almost half of the cases were caused by *S. pneumoniae* and more than half of the patients showed radiological signs of complications (bronchopulmonary fistula in 29% and necrotising pneumonia in 26%). Hence, this unprecedentedly high proportion of patients undergoing surgical management may have resulted from exceptionally severe cases but also late interventions (Table S1, Figs. 1 and 2). Fibrinolytics were frequently used from 2008 to 2016, but thereafter, their use decreased. This can be explained by the ongoing supply shortages but also by the evidence that fibrinolytics are not associated with patient outcomes [14, 26, 27]. We observed a continuous but not significant decline in LoS, except for the outlier period from 2016 to 2020, presumably explained by the aforementioned high proportion of patients managed with surgery (29%) (Figs. 1 and 2). Days on intravenous antibiotics, however, declined significantly over time. This likely reflects increased efforts in antibiotic stewardship effected through a new in-house PPE treatment guideline established in 2010 advocating for 10–14 days of antibiotic treatment compared to international guidelines recommending 2–4 weeks of treatment [8, 9] or other hospitals’ common practice [28, 29]. 

With regard to pleural drainage, we made our decision based on a multidisciplinary team discussion involving paediatric pneumologists, paediatric surgeons, and paediatric infectious diseases specialists. Apart from imaging, we mainly relied on the severity of respiratory or cardiovascular distress and the absence of clinical improvement. This approach usually resulted in a stepwise process, and surgical interventions were applied when antibiotics with or without a pleural drain alone had failed. Hence, patients requiring surgery were later in the course of the disease and comprise a selection of more complicated cases. This may be even more applicable in recent times because our numbers show a steady and significant increase of the interval from admission to surgery (Fig. 2). Therefore, even though the intervention per se may slow down recovery for several days, it can still have a beneficial net effect and numbers referring to outcome parameters like LoS have to be interpreted cautiously. The evidence from the available literature and guidelines, however, is conflicting. While some data shows that those children whose disease course is more complicated and whose PPE is clinically more relevant have a higher risk for surgical interventions and, therefore, are more likely to spend longer time in hospital [30, 31] a systematic review and meta-analysis by Elviro et al. showed the contrary, i.e., that a pleural drain alone was associated with a longer LoS compared to other invasive treatment modalities [23, 25]. The aspect that patients requiring a surgical intervention suffered from a more complicated clinical course is supported by our data showing pulmonary damage in up to every fifth patient but significantly more often in patients after surgery compared to a chest drain alone. Recent data from the United Kingdom reported bronchopleural fistula at 33% and speculated to be associated with *S. pneumoniae* serotype 3 [30, 32]. Additionally, we found that drains were significantly shorter in situ in patients without surgical interventions, LoS was five days, and intravenous antibiotic treatment duration was three days shorter. On the other hand, patients with PPE and VATS/thoracotomy showed similarly elevated inflammatory markers, no differences in the presence of septic or toxic shock, and same proportions of patients requiring inotropic support or intubation compared to patients with pleural drainage alone. Considering the aforementioned aspects, like the stepwise approach and the finding that all radiological signs of lung damage occurred more frequently in patients with VATS/thoracotomy, we believe that patients who underwent VATS/thoracotomy were more severely affected than those who were treated with a pleural drainage alone. Our retrospective data, however, do not allow us to prove causality but especially in times of austerity measures in the healthcare system, five hospitalisation days are relevant, and further research should be done to untangle the exact reasons for these differences. Figures 1 and 2.

Over 20 years, two-thirds of microbiologically confirmed PPE was caused by *S. pneumoniae*, except for the last 4 years, when numbers were even, explained by the upsurge of *S. pyogenes* PPE, in line with the Europe-wide outbreak of invasive group A streptococcal infections [33, 34]. This outbreak allowed for sufficient case counts to compare clinical characteristics between the two groups, while not much literature is available, so far [35]. Patients with *S. pyogenes*-associated PPE were significantly more often exhibiting signs of septic or toxic shock and were admitted to PICU with vasoactive support at 66% and 39%, respectively, compared to patients with *S. pneumoniae*-associated PPE. Such elevated proportions of critically ill patients with *S. pyogenes-*PPE have also been reported by Lees et al. with PICU admission rates of around two-thirds and a need for inotropes of 35.7% [29]. A group from Canada reported a PICU admission rate of 41.7% [36], and French data showed even higher PICU admission rates (86%) [28]. It is well known that the onset of streptococcal toxic shock syndrome is rapid [28], and in our cohort, only two of 31 patients were prescribed antibiotic treatment prior to admission compared to 25 of 67 patients with *S. pneumoniae*-associated PPE. The presence of scarlet fever was common in patients (35%) with *S. pyogenes*. In contrast, patients with *S. pneumoniae* compared to those with *S. pyogenes* were significantly more at risk of developing pulmonary damage (68% vs. 23%). Considering differences in presentation, we believe it is important to rapidly identify the aetiology of PPE. Supposedly, patients with *S. pyogenes* infection would benefit from single pleural drainage, mainly for diagnostic purposes, and long-term radiological follow-up might be unnecessary. However, these patients should be monitored very closely for signs of septic or toxic shock during the early phases of the disease. Patients with *S. pneumoniae* PPE, on the other hand, should be informed about the higher risk for lung damage, and in these cases, fibrinolytics should be used even more cautiously. The proportion of patients in whom no pathogen could by identified declined over the course of the study. Since broad-range PCR was available and used throughout the entire study period, we believe this decrease is mainly related to increased awareness and use of PCR technology, in addition to an incline in chest drainage alone approach, rather than improved technologies.

This study has two main strengths. First, the long period of 20 years and the high number of included patients. Second, this data collection comprises laboratory values, imaging studies, and information on interventions that improve understanding of PPE. The most important limitation of the study is the retrospective observational study design with the inherent risk of missing cases. A potential explanation for why cases were lower from 2004 to 2008 because the electronic patient file was only introduced in 2008, and presumably before then, not all cases were identified. Furthermore, important information such as vaccination status, virology testing, or serotype identification was not systematically available. Next, this study was set up as a single-centre study, hence, in-house expert opinion and guidelines determine study findings. The comparison of *S. pyogenes* and *S. pneumoniae* is hampered because half of the cases caused by *S. pyogenes* occurred during the *S. pyogenes* outbreak in 2022/2023, when other factors might have additionally impacted the clinical course and influenced our results. Lastly, for subanalyses and multiple logistic regressions, numbers were small, resulting in broad confidence intervals.

To summarise, the management of patients with PPE remains difficult, and currently available research data do not allow for a unanimous interpretation. Standardised, multidisciplinary management and therapy guidelines are needed, specifically addressing management at an early stage. Respecting the credo ‘do no harm’, we believe that conservative treatment or chest drain only should be prioritised until we better understand when and what surgical intervention improves patient outcomes, such as shortening LoS, improving antibiotic stewardship, reducing pulmonary damage, and is cost-effective. Focusing on rapid pathogen identification by using rapid antigen detection testing in pleural fluid samples could be one possibility to select a more pathogen-specific management approach [37]. Overall, to better answer and understand relevant factors and mechanisms, a randomised controlled multi-centre interventional trial would be needed, acknowledging that the set-up of such a trial would be very challenging. The results of this study can help frame questions for such studies, but also might inform guideline development.

## Supplementary Information

Below is the link to the electronic supplementary material.

Supplementary Material 1

## Data Availability

Data are available on reasonable request. Deidentified participant data will be shared on reasonable request unless the request is conflicting with ongoing or planned analyses. Requests need to be addressed to the corresponding author and will require approval by all co-authors.
