# Supplementary material for: Paediatric parapneumonic effusion – a twenty-year clinical narrative
Source: Infection. 2025 Oct 14;54(1):287–97. doi: 10.1007/s15010-025-02662-1 (PMC12864278; doi:10.1007/s15010-025-02662-1)
Supplement: Supplementary file 1 — Supplementary Material 1 [file 15010_2025_2662_MOESM1_ESM.docx]

**PAEDIATRIC PARAPNEUMONIC EFFUSION – A TWENTY-YEAR CLINICAL NARRATIVE**

Leonie Bregy^1^, Philipp KA Agyeman^1^, Andrea Duppenthaler^1^, Elisabeth Kieninger^2^, Matthias Horn^3^, Jonathan Juzi^4^, Dietmar Cholewa^4^, Carmen Casaulta^2^, Matthias V Kopp^3^, Christoph Aebi^1^, Nina Schöbi^1^

**Content**

| Table S1 | Time trends of clinical variables in patients with parapneumonic effusion undergoing chest interventions, Department of Pediatrics, Bern University Hospital, 2004-2024 |
| --- | --- |
|  |  |
|  |  |
|  |  |
|  |  |
|  |  |

| Table S1. Time trends of clinical variables in patients with parapneumonic effusion undergoing chest interventions, Department of Paediatrics, Bern University Hospital, 2004-2024 | | | | | | | |
| --- | --- | --- | --- | --- | --- | --- | --- |
|  |  |  |  |  |  |  |  |
|  |  |  |  |  |  |  |  |
|  | Period (epidemiological years) | | | | |  |  |
|  | 2004-2008^1^ | 2008-2012^1^ | 2012-2016^1^ | 2016-2020^1^ | 2020-2024^1^ |  | p value (univariate)^3^ |
| Cases (n) | 30 | 32 | 43 | 31 | 37 |  |  |
| Female sex | 14 (47) | 14 (44) | 21 (49) | 14 (45) | 15 (41) |  | 0.962 |
| Age (years) | 3.7 [2.3-7.4] | 4.4 [2.4-7.2] | 4.1 [2.7-6.4] | 4.7 [3.2-6.0] | 3.9 [2.0-5.6] |  | 0.882 |
|  |  |  |  |  |  |  |  |
| Secondary referral from peripheral hospital | 8 (27) | 14 (42) | 18 (42) | 10 (32) | 16 (43) |  | 0.522 |
| Duration of illness before primary admission (days) | 5.0 [4.0-7.0] | 5.0 [3.0-7.0] | 5.0 [3.0-7.0] | 5.0 [3.0-7.0] | 5.0 [3.0-7.0] |  | 0.951 |
| Prior oral antibiotic therapy | 6 (20) | 12 (36) | 15 (35) | 8 (26) | 9 (24) |  | 0.466 |
| Comorbidity, any | 3 (10) | 4 (12) | 15 (35) | 5 (16) | 6 (16) |  | 0.043 |
|  |  |  |  |  |  |  |  |
| Clinical scarlet fever | 2 (6.7) | 4 (12) | 2 (4.7) | 3 (9.7) | 3 (8.1) |  | 0.789 |
| Septic shock or toxic shock syndrome | 4 (13) | 5 (15) | 1 (2) | 2 (6) | 7 (19) |  | 0.125 |
|  |  |  |  |  |  |  |  |
| CRP, admission (mg/L)^2^ | 300 (128) | 267 (83) | 246 (104) | 261 (84) | 272 (106) |  | 0.282 |
| WBC, admission (G/L)^2^ | 17.9 (12.3) | 13.6 (8.5) | 18.3 (8.3) | 17.8 (7.7) | 16.4 (10.3) |  | 0.261 |
| Platelet count, highest (G/L)^2^ | 747 (272) | 813 (268) | 783 (311) | 807 (281) | 971 (434) |  | 0.043 |
| Bacteraemia | 8 (27) | 5 (15) | 3 (7) | 2 (7) | 2 (5) |  | 0.036 |
|  |  |  |  |  |  |  |  |
| Chest sonography performed | 25 (83) | 30 (91) | 42 (98) | 30 (97) | 36 (97) |  | 0.075 |
| Chest computerised tomography performed | 12 (40) | 6 (18) | 15 (35) | 14 (45) | 10 (27) |  | 0.112 |
|  |  |  |  |  |  |  |  |
| Radiologic evidence for |  |  |  |  |  |  |  |
| Pneumatocele or bullae | 12 (40) | 9 (27) | 14 (33) | 9 (29) | 9 (24) |  | 0.723 |
| Seropneumothorax | 8 (27) | 7 (21) | 18 (42) | 14 (45) | 6 (16) |  | 0.031 |
| Bronchopleural fistula | 2 (7) | 2 (6) | 6 (14) | 9 (29) | 9 (24) |  | 0.039 |
| Intrapulmonary abscess | 2 (7) | 3 (9) | 2 (5) | 1 (3) | 5 (14) |  | 0.489 |
| Necrotising pneumonia | 2 (7) | 2 (6) | 3 (7) | 8 (26) | 9 (24) |  | 0.020 |
| ≥1 of the 5 criteria listed above | 16 (53) | 13 (41) | 24 (56) | 18 (58) | 15 (41) |  | 0.411 |
|  |  |  |  |  |  |  |  |
| Pathogen identified |  |  |  |  |  |  |  |
| *S. pneumoniae* | 12 (40) | 12 (36) | 12 (28) | 15 (48) | 16 (43) |  | 0.448 |
| *S. pyogenes* | 5 (17) | 4 (12) | 2 (4.7) | 6 (19) | 14 (38) |  | 0.003 |
| Other | 2 (7) | 3 (9) | 5 (12) | 2 (7) | 1 (3) |  | 0.641 |
| No pathogen identified | 11 (37) | 13 (39) | 23 (53) | 8 (26) | 6 (16) |  | 0.008 |
|  |  |  |  |  |  |  |  |
| Antibiotic therapy, duration (days) |  |  |  |  |  |  |  |
| Intravenous | 15.0 [14.0-19.8] | 14.5 [13.0-15.3] | 14.0 [10.0-18.5] | 14.0 [12.0-19.5] | 11.0 [8.0-15.0] |  | 0.002 |
| Intravenous and oral step-down therapy combined | 18.0 [14.0-24.0] | 15.0 [14.0-19.0] | 15.0 [14.0-24.0] | 15.0 [14.0-22.0] | 15.0 [14.0-22.0] |  | 0.542 |
|  |  |  |  |  |  |  |  |
| Chest interventions |  |  |  |  |  |  |  |
| Chest drain only | 6 (20) | 20 (61) | 28 (65) | 18 (58) | 24 (65) |  | 0.001 |
| Chest drainage duration (days) | 5.5 (4.0-7.0) | 5.0 [3.0-5.0] | 5.0 [4.0-8.0] | 5.0 [4.0-12.5] | 3.0 [2.0-7.5] |  | 0.071 |
| Fibrinolytic therapy | 1 (3.3) | 22 (67) | 24 (56) | 6 (19) | 4 (11) |  | <0.0001 |
| VATS | 22 (73) | 11 (33) | 11 (26) | 4 (13) | 13 (35) |  | <0.0001 |
| Thoracotomy | 2 (7) | 1 (3) | 4 (9) | 9 (29) | 0 |  | 0.0005 |
|  |  |  |  |  |  |  |  |
| Length of stay (d) | 16.5 [14.0-23.5] | 15.0 [13.0-16.0] | 14.0 [10.0-21.0] | 16.0 [13.0-24.0] | 12.0 [9.0-24.0] |  | 0.046 |
| PICU admission | 19 (63) | 15 (46) | 16 (37) | 15 (48) | 16 (43) |  | 0.277 |
| Inotropes | 2 (6.7) | 4 (12) | 3 (7.0) | 3 (9.7) | 8 (22) |  | 0.250 |
| Intubation | 2 (6.7) | 3 (9) | 5 (12) | 4 (13) | 8 (22) |  | 0.406 |
|  |  |  |  |  |  |  |  |
| Rehospitalisation for complications of PPE | 2 (7) | 0 | 3 (7) | 1 (3) | 5 (14) |  | 0.204 |
| ^1^ n (%) or median [interquartile range] are shown except for variables marked with superscript "2" | | | |  |  |  |  |
| ^2^ mean and standard deviation (SD) are shown for this variable | |  |  |  |  |  |  |
| ^3^ 2x5 contingency table, one-way analysis of variance (ANOVA), or Kruskall-Wallis test were used as appropriate | | | | |  |  |  |
